# Sex/gender differences in cognition, neurophysiology, and neuroanatomy

**DOI:** 10.12688/f1000research.13917.1

**Published:** 2018-06-20

**Authors:** Lutz Jäncke

**Affiliations:** 1 Division of Neuropsychology, Institute of Psychology, University of Zurich, Zurich, Switzerland; 2 University Research Priority Program (URPP) “Dynamic of Healthy Aging”, University of Zurich, Zurich, Switzerland

**Keywords:** Sex/gender differences, brain anatomy, brain function, cognition

## Abstract

In this mini-review, I summarize and interpret the current status of sex/gender differences in terms of brain anatomy, brain function, behavior, and cognition. Based on this review and the reported findings, I conclude that most of these sex/gender differences are not large enough to support the assumption of sexual dimorphism in terms of brain anatomy, brain function, cognition, and behavior. Instead, I suggest that many brain and cognitive features are modulated by environment, culture, and practice (and several other influences). These influences interact with the menstrual cycle, the general hormone level, and current gender stereotypes in a way that has not yet been fully understood.

## Introduction

For centuries, humans have been fascinated by the idea of psychological gender differences, many believing that these differences are both large and biologically determined. In this context, it has been argued that such differences are determined by genetic and hormonal influences affecting brain anatomy or brain function or both. Often, it has been speculated that these biological differences are a consequence of sex-/gender-specific evolutionary processes that ultimately determine sex-/gender-specific roles in human societies. This research has also been strongly influenced by animal research, where it is much easier than it is in humans to study genetic differences in terms of sex/gender, including at the molecular, hormonal, and neurophysiological levels ^1, 2^. However, it is not a simple endeavor to transfer results and interpretations from animal research to explain human behavior and cognition, since there are still some substantial differences between humans and other animals. One major difference is that the brain of humans is different in many respects from the brain of most other animals, although the human brain comprises the same neurons as even simpler constructed animals. The human brain comprises the largest number of neurons compared with all other animals in absolute terms ^3^. In addition, it is characterized by extreme, and in the animal kingdom unprecedented, interconnectivity that provides the necessary basis for the computation and storage of information, which is necessary for human learning and culture ^4^. This huge neural network is also significantly plastic and can be shaped by individual experience and practice ^5– 8^.

With respect to understanding psychological gender differences, individual learning experiences, culture, gender stereotypes, gender equity, and biosocial interaction are of the utmost importance ^9^. In addition, recent brain plasticity research provides evidence that gender brain differences might also be shaped by experience, education, and culture or a combination of these. Thus, these new insights into the influences on behavior, brain anatomy, and brain function shed new light on the often-reported gender differences. In this mini-review, I summarize and discuss these new findings and ideas.

In the scientific literature and the popular press, the terms sex and gender differences are often used interchangeably. However, they convey different meanings. The term sex is used mostly to group people into females and males on the basis of an individual’s reproductive system and of secondary sexual characteristics. Gender refers to the social roles based on the sex of the person or personal identification of a person’s own gender. Since it is not clear whether the brain and behavioral differences that I discuss in this mini-review are sex- or gender-based, I use the term sex/gender differences throughout the text.

## Sex/gender differences versus sex/gender dimorphism

As McCarthy and Konkle ^2^ and Joel and Fausto-Sterling ^10^ elegantly emphasize, there is an urgent need to carefully distinguish between the terms sexual dimorphism and sex differences. They argue that one should use the term sexual dimorphism only for those aspects of differences that come in two distinct forms. As an example, they suggest male and female genitalia or X and Y chromosomes that appear in just two forms (with some exceptions). With respect to sex/gender differences, it is obvious that even very large sex/gender brain and behavioral differences are not dimorphic since the reported features overlap too much when the feature distribution for males and females is considered. They also point out that true sexual dimorphism is extremely rare in the human brain (but also in terms of behavior and cognition). As examples, they mention the very large sex/gender differences for the intermediate nucleus (InM) of the hypothalamus, which is on average twice as large in males as it is in females. However, in about a third of the cases, males and females demonstrate InMs of the same size. Thus, terms such as “female brains” or “male brains”, which are frequently used in popular writing, should not be used since it is difficult or even impossible to identify typical and dimorphic features that justify a clear sex/gender classification. However, Joel and Fausto-Sterling ^10^ even argue that a particular brain might comprise one feature which is statistically more typical of females while another feature might be more typical for males. In this context, it could be possible that different brain features are related in a kind of compensatory relationship. To explain this, they report sex-environment interactions shown in animal research by referring to a study by Reich *et al.* ^11^, which illustrates that three weeks of mild stress reversed a sex difference in the density of CB1 receptors in rats’ dorsal hippocampi. Thus, an anatomical sex difference is reversed because of a particular environmental influence. An analogous finding has been reported in the context of aging research. Several studies have shown that prefrontal activity during cognitive performances tends to be less lateralized in older adults than in younger adults more or less independent of sex/gender (hemispheric asymmetry reduction in older adults [HAROLD] model) ^12^. This age-related hemispheric asymmetry reduction is thought to have a compensatory function. This asymmetry reduction is of interest here, since an asymmetry reduction is often interpreted as a typical feature of a “female brain” thought to indicate advantageous “female” bilateral processing.

In order to make the distinction between sex differences and sexual dimorphism slightly clearer, it is useful to refer to the frequently used effect size measure proposed by Cohen (Cohen’s d) ^13^. Cohen’s d reflects the normalized difference between the sexes with respect to a particular measure. This effect size assesses the magnitude of difference in two-group designs. For sex/gender differences, the formula is d = (MM − MF) / SW, where MM is the mean score for males, MF is the mean score for females, and SW is the within-group standard deviation. Thus, the d statistic represents the difference between two means normalized to the common standard deviation. The advantage of this effect size measure is that it is independent from sample size, applicable to different measures, and easily combinable across different studies.

Cohen ^13^ suggests categorizing the obtained d values into small (0.20), moderate (0.50), and large (0.80) effects. Let us assume that we examine the performance in a particular cognitive task in 100 men and 100 women, whereby we obtain different performance measures for all subjects which will distribute normally and separately for men and women. Thus, we obtain two overlapping distributions containing the performance values for men and women. Both distributions will have a mean, and the differences between both means can be expressed as the d statistic. In the event of normal distributions, we can calculate, on the basis of the d statistic, several further interesting statistics that help us to understand the meaning of the d value. With a d of 0.5 (which is approximately the average effect for a common sex difference in the mental rotation performance of 3D objects, typically one of the most consistent and largest cognitive sex/gender differences; see “Cognition and emotion” section below), 69% of the men’s performance will be above the mean of the women’s performance (calculated on the basis of Cohen’s U3). In addition, 80% of the two groups will overlap, and there is a 64% chance that a man picked at random will have a higher score than a woman picked at random (probability of superiority). Now let us assume d = 1.4, which is roughly the typical d for sex/gender differences of total brain volume ^14^ (in the large-scale study by Jäncke *et al.* ^15^, the sex/gender difference is much smaller [d = 1.1]); here, there is an overlap for the male and female distributions of more than 48%. A total of 92% of the male group will be above the mean of the female group (Cohen’s U3), and there is an 84% chance that a person picked at random from the male group will have a higher brain volume than a person picked at random from the female group (probability of superiority).

In summary, even in the case of a very large effect size of d = 1.4, it is difficult or even impossible to draw conclusions on single subjects, since too many men and women fall into the same range. Although this can be interpreted as a sex/gender difference, this large difference is not a typical sexually dimorphic trait. In the following sections of this mini-review, I describe typical sex/gender differences for brain and behavioral measures and also discuss some environmental factors influencing or modulating (or both) these sex/gender differences.

## Cognition and emotion

Since the beginning of scientific research into sex/gender differences with respect to cognition and emotion, many studies on these topics have been published. These results have been summarized in older and influential reviews which have concluded that sex/gender differences in verbal, spatial, and mathematical abilities would be well established, with males scoring higher on spatial and mathematical tests and females higher on verbal tests ^16, 17^. These studies have also inspired several popular books on sex/gender differences which are regularly bestsellers ^18– 20^. However, several meta-analyses of published and unpublished scientific work on these sex/gender differences paint a different picture, suggesting that males and females are much more similar in terms of cognitive abilities and emotions than previously anticipated ^9, 21– 23^.

Comprehensive meta-analyses on sex/gender differences for many cognitive tasks and psychological tests have been undertaken by Janet S. Hyde. In her first large-scale meta-analysis ^22^, she analyzes sex/gender differences for 124 psychological variables, comprising performance measures in mathematical, verbal, perceptual, and motor tasks. In addition, she reports effect sizes for measures related to personality, aggression, sexual behavior, leadership, social behavior, life satisfaction, moral reasoning, delay of gratification, cheating behavior, and job-related issues. The most important finding of her study is that 78% of the effect sizes are in the close-to-zero range or small (0 < d > 0.35). Thus, for the majority of the measured psychological variables, she identifies practically no, or only small, sex/gender differences.

The largest sex/gender differences are found for motor performance, particularly for measures such as throwing velocity (d = 2.18) and throwing distance favoring men. Close-to-zero differences were found for mathematical and verbal abilities, both psychological domains for which strong sex/gender differences have been proposed and reported in single studies. Only for the mental rotation of 3D objects have substantial performance differences been identified, with men and boys outperforming women and girls (d = 0.51–0.73) ^24, 25^. A recent larger non-meta-analytical study comprising more than 1,000 subjects identifies a d ranging between 0.72 and 0.91; however, it also shows the strong influence of academic background, educational level, and stereotyping ^26, 27^ (see below in this section). It must also be taken into account that sex/gender differences are probably overestimated simply because we can assume that many researchers recruit participants by asking them to take part in a study on sex/gender differences. This will lead to stereotypic priming of the participants.

An important aspect for this research is that sex/gender differences for at least two domains for which mostly sex/gender differences have been reported (for example, mathematical and verbal skills) are influenced by culture, education, and training. For example, a recent meta-analysis comprising data from 242 studies (!) conducted between 1990 and 2007 ^28^ containing data from 1.2 million (!) children and adults reveals no sex/gender differences in math performance with a d = 0.05, thus confirming previous meta-analyses. A similar picture emerges for verbal abilities, for which all meta-analyses reveal close-to-zero effect sizes for sex/gender differences.

Overall, these meta-analyses indicate that females have reached parity with males in math and verbal ability performance today, although there are variations in this pattern as a function of several factors, such as nation and culture. The influence of culture, education, gender equity, and gender stereotype on these abilities has become a major focus in this research area. Owing to the limited space in the context of this mini-review, I cannot review all work published so far. However, large international studies have uncovered substantial cross-cultural variations in cognitive sex/gender differences, challenging the notion of universal male advantages in mathematics and female advantages in verbal abilities ^29, 30^. These findings have also been critically described in recent reviews ^9, 21, 23, 30^.

The strongest cognitive sex difference which has been observed so far has been found for the mental rotation of 3D objects with moderate to large effect sizes (d = 0.51–0.73). These differences favoring males have mostly been identified controlling for educational and cultural background. However, several studies have shown that performance in mental rotation strongly depends on practicing spatial functions as well as on educational and cultural background ^26^. For example, practicing mental rotation or spatial tasks increases the performance in mental rotation in males and females. In addition, students from engineering, mathematical, and science faculties consistently outperform students from arts faculties. Even priming academic background can implicitly prime gender-specific effects with negative consequences for women’s cognitive performance, particularly with respect to mental rotation ^27^.

Cultural influences seem to have a strong effect on cognitive performance, especially in terms of mental rotation. This is demonstrated in an influential paper ^31^ in which the authors examine spatial abilities (using a spatial puzzle) in nearly 1,300 participants and show that the sex difference in spatial abilities disappears in participants from a matrilineal society but that the sex difference favoring males is still present in participants from a patrilineal society. They also demonstrate that large parts of this effect must be due to differences in education. The authors argue that these results indicate the role of nurture in inducing sex/gender differences in cognitive abilities, since the participants experienced the used spatial task for the first time, both societies have the same means of subsistence, and they share the same genetic background.

Interactions between gender stereotypes and hormone levels are also possible. Only a few studies have investigated the combined effects of sex hormones and gender stereotypes. An important paper in this context has been published by Hausmann *et al.* ^32^. They examined a relatively large sample of men and women using a battery of sex-sensitive cognitive tasks (mental rotation, verbal fluency, and perceptual speed) and controlled for levels of testosterone during testing. In addition, they activated gender stereotypes using questionnaires referring to the cognitive tasks prior to the experiment. The control group received a questionnaire with gender-neutral content. The authors identified that the male superiority in mental rotation performance in the entire sample was driven mainly by the gender-stereotyped group. There was in fact no gender/sex difference in mental rotation for the control group. Another interesting finding was that testosterone levels in the gender-stereotyped group were 60% higher than those of male controls. This study elegantly demonstrates that sex hormones strongly interact with gender stereotypes and at the end influence specific cognitive abilities.

Whereas older studies on sex/gender differences with respect to cognition have mostly neglected the influence of menstrual cycle on cognition, several studies published in the last 20 years have clearly demonstrated the influence of menstrual cycle on cognition. A typical study design in this context is to examine cognitive performance time-locked to the follicular (low-progesterone) and luteal (high-progesterone) phases in women. With such designs, it has been shown that different progesterone and estradiol levels are substantially linked to attention, executive functions, spatial navigation, and functional asymmetries ^33– 39^.

A further more naturalistic study uncovers no differences between males and females in terms of a major verbal ability ^40^. Using an audio recorder attached to the participants that recorded ambient sounds for several days, the researchers extrapolated the number of spoken words per day and conclude that women and men both spoke about 16,000 words per day. Thus, the often-mentioned argument that women speak more than men is challenged by this elegant scientific experiment.

With respect to emotions, empathy, moral judgment, and social behavior, measures of sex/gender differences are very small or even disappear when studying larger samples or using more objective measures than self-report ones. This has already been shown in the first comprehensive review by Hyde ^22^. However, typical gender stereotypes such as that women are more empathetic, caring, emotional, sensitive, and moral than men are prevalent in our culture. Studies supporting these gender stereotypes have been obtained mostly through self-report questionnaires ^41– 43^, which may be strongly biased by gender-relevant social expectations. Sex differences are often absent or very small in relevant experimental tasks using physiological measures ^44, 45^ and when studies are conducted using large samples ^46^.

In conclusion, males and females are more similar in terms of cognitive functions and emotions than previously anticipated. Cultural background, education, gender equity, gender stereotypes, practice, and hormone levels have substantial influences on cognition and emotion. Thus, there is an increasing amount of scientific findings supporting the gender similarity hypothesis first proposed by Janet Hyde ^22^.

## Brain anatomical differences

It is often argued that sex hormones present during critical developmental periods (for example, *in utero*, shortly after birth, or during puberty) might induce permanent effects on brain organization and brain activation. The most famous hypothesis of this type is proposed by Geschwind, Behan, and Galaburda (the so-called Geschwind-Behan-Galaburda theory) ^47– 49^. They argue that both hemispheres mature differently because of differences in circulating testosterone levels. For example, increased testosterone levels during fetal development reduce the rate of left-hemispheric development and stimulate an increased growth of posterior right-hemispheric regions, ultimately resulting in an altered inter-hemispheric balance. Geschwind, Behan, and Galaburda suggested many additional consequences induced by different testosterone levels, which cannot be described here (for a summary, see 50). For the scope of this mini-review, it is important to note that they suggest very specific anatomical and functional sex/gender differences. At that time, the methodology for studying sex-specific neuroanatomical and neurophysiological differences was not as advanced as it is today. Thus, most studies of that time used non-invasive behavioral measures to examine this theory. Based on these data, Bryden *et al.* ^51^ come to the conclusion that this theory is not well grounded and that “psychologists and physicians have more useful things to do than to carry out further assessments of the model”. However, others argue that it is too early to dismiss this theory only on the basis of behavioral data and that more careful neuroscientific and neurological studies should be carried out ^52^.

A further line of evidence proposes that female and male brains demonstrate different patterns of intra-hemispheric and inter-hemispheric connectivity. Early research in this area suggests stronger and more effective inter-hemispheric connections in women as indicated, for example, by larger cross-sectional corpus callosum (CC) areas (representative of the number of transcallosal fibers) ^53^. However, many studies have failed to replicate this finding ^54^, particularly when morphological sex/gender differences of the CC are related to total brain size ^55^. This similarity with respect to inter-hemispheric connectivity corresponds well to the apparent functional laterality similarity between sexes ^56^.

This issue has, however, received new interest due to a recent study that reports greater within-hemispheric connectivity in men and greater between-hemispheric connectivity in women on the basis of diffusion tensor imaging and graph analytical approaches ^57^. In addition, this paper has led to heated discussions because the findings have been interpreted by the authors as evidence that female brains are designed to facilitate communication between analytical and intuitive processing modes. However, a further study compares the anatomical connectivity measures between men and women for groups of men and women with similar brain sizes and identifies small or non-existent sex/gender differences ^58^. Thus, sex/gender differences in terms of inter-hemispheric connectivity could also depend on brain size differences.

Most studies report sex/gender differences for many brain anatomical features with moderate to strong sex/gender differences. For example, total brain volume, as well as gray matter and white matter volumes, reveals sex/gender differences ^14, 15, 59^ with d values ranging between 1 and 1.49 ^14, 15^. The sex/gender differences for subcortical volume measures (for example, basal ganglia and thalamus) are much smaller, and the range of d is 0.31 to 1.03. When brain size is corrected for, these sex/gender differences nearly disappear ^15^.

In this context, it is interesting to note that brain anatomical sex/gender differences have been reported even when brain size is controlled for. For example, Luders *et al.* ^60^ report greater gyrification in women than men in frontal and parietal regions. This implies more cortical surface area, which may offset gender differences in brain volume. Using a new technique to identify anatomical regions of interest, Kurth *et al.* ^61^ also identify significantly larger gray matter volumes in females compared with males for BA 44 and BA 45 bilaterally, which are brain areas known to be involved in controlling verbal functions. In addition, there are several papers demonstrating brain maturation differences, with girls maturing slightly earlier ^62– 65^. However, it has also been shown that the uncovered sex-/gender-specific brain maturation profiles are modulated by several further issues (for example, intelligence and psychiatric diseases ^63, 66^). Thus, it will be interesting to see whether future well-conducted studies uncover that nutrition, education, cultural background, stimulation, or other factors modulate brain development more or less independently from sex.

Although consistent gender differences are repeatedly reported and documented, Joel *et al.* ^67^ argue that these differences are not suitable for establishing a sexual dimorphism in terms of brain anatomy, mostly because the anatomical parameters for men and women overlap far too much. There are also too few women and men who demonstrate exclusively male or female brain characteristics. Rather, the authors assume that male and female brains are both composed of male and female brain features (mosaics). Thus, typical female or male brains do not exist. An important aspect in this context is the fact that it has been shown that brain anatomy (and brain size) strongly depends on nutrition, obesity, diet, culture, famine history, age, education, cardiovascular risk factors, and skill ^5, 7, 8, 68– 80^.

From the above-mentioned findings, we can summarize that on average there are moderate to strong brain anatomical sex/gender differences (which are substantially smaller for subcortical structures). These brain volume differences are also associated with sex/gender differences in terms of inter- and intra-hemispheric anatomical connectivities. Nevertheless, even these sex/gender differences are not compelling enough to support the hypothesis of an existing sexual dimorphism in brain anatomy. Besides the fact of strong overlaps between male and female distribution, it has to be considered that brain anatomy is substantially affected by environmental influences. Most importantly, however, is that the relationship between brain anatomical measures as mentioned above and cognition, behavior, and emotion is currently not clear. We must therefore be very careful if we explain gender differences in cognition, emotion, and behavior on the basis of brain anatomical findings.

## Brain activation differences

An issue often raised is that brain activations during the performance of specific cognitive tasks are associated with characteristic sex/gender differences. Typically, it is argued that women show a more bilateral activation pattern, for example during the processing of verbal information. This is demonstrated in a widely cited paper by Shaywitz *et al.* ^81^, who report bilateral hemodynamic responses in frontal language areas in females during verbal monitoring tasks. This activation pattern has not been replicated in subsequent studies from other groups employing many more subjects than the study by Shaywitz *et al.* ^82^. Even meta-analytical studies summarizing published functional magnetic resonance imaging (fMRI) studies on that topic reveal no consistent sex/gender differences with respect to cortical activation differences during language tasks ^83, 84^. However, one has to keep in mind that fMRI studies usually rely on small sample sizes and that the paradigms used in (verbal) fMRI studies vary considerably.

Substantial sex/gender differences in emotional responses and perception have been reported in several psychological and psychophysiological studies. Mostly, it has been shown that women respond more strongly to negative emotional stimuli than do men. This difference has often been linked to an increased risk of depression and anxiety disorders in women. A recent meta-analysis summarizing the published neuroimaging studies on that topic addresses this issue ^85^. The authors identify a couple of sex/gender differences in terms of brain activation in several brain systems. The majority of these differences favoring women are observed for negative emotions, whereas the majority of the sex/gender differences favoring men are observed for positive emotions. This valence specificity is particularly evident for the amygdala. For negative emotions, women exhibited greater activation than did men in the left amygdala as well as in other regions, including the left thalamus, hypothalamus, mammillary bodies, left caudate, and medial prefrontal cortex. In contrast, for positive emotions, men exhibited greater activation than did women in the left amygdala as well as greater activation in other regions, including the bilateral inferior frontal gyrus and right fusiform gyrus.

Nevertheless, the study of sex/gender differences in terms of brain activation is still far from complete. A major issue in this research area is that most of these studies have neglected the influence of the menstrual cycle on brain activation and behavior. One of the first is a study in which the female subjects, who did not use oral contraceptives, were scanned twice, once during their menses and once on the 11 ^th^ or 12 ^th^ day of the menstrual cycle ^86^. In addition, the authors examined males for further comparison. All subjects performed a word-stem completion, a mental rotation, and a simple motor task while hemodynamic responses were measured using fMRI. The authors conclude that the menstrual cycle hormones influence the overall level of cerebral hemodynamics. No differences were observed between male and female subjects during the low-estrogen phase. During the verbal and spatial tasks (but not during the motor task), blood estrogen level had a profound influence on the spatial extent of cortical activation. Female brains under estrogen showed a marked increase in hemodynamic responses in those cortical areas involved in controlling the particular cognitive task. Recent studies have confirmed these results and demonstrated the substantial influence of sex hormone levels on brain activation during task performance ^87– 91^ (for a stable trait-like resting state network independent from the menstrual cycle, see 92). Even resting state activity (a state during which no cognitive task is processed) is associated with brain activations and functional network features which substantially vary as a consequence of sex hormone level fluctuations during the menstrual cycle ^90^. Thus, when men and women are compared in terms of brain activation and task performance in psychological tasks, there is an urgent need to consider the particular phase of the menstrual cycle and the associated hormone levels.

Besides these hormone-level influences on brain activations, there is a wealth of literature demonstrating practice- and skill level-dependent brain activations during task performance and resting state ^93– 95^. Thus, one can conclude that sex/gender differences in terms of brain activations are strongly influenced by education, practice, skill level, and hormone levels. Possible sex/gender differences can be enhanced, diminished, or even eliminated because of these influences. Nevertheless, future studies should carefully control for these influences when designing experiments to delineate “true” sex/gender differences.

## Conclusion and outlook

The study of sex/gender differences attracts the attention of a considerable amount of research in addition to the public media, politicians, and laypersons. Many use neuroscientific sex/gender differences to explain and partly justify social and behavioral differences. However, the research of the past 50 years and particularly of the last 10 years has shown that sex/gender differences in terms of cognitive functions are less clear than previously assumed. Both sexes are more similar in respect to many psychological functions, and it is also now clear how strong the influence of culture and social stereotypes is. In addition, the sex/gender differences in brain anatomy and brain function are less clear. There are some relatively strong but also many moderate or even weak sex/gender differences in terms of brain anatomy and brain function. These differences are not large enough to support a clear sexual dimorphism. Thus, there is no strong evidence available supporting the existence of a typical “female” or “male” brain.

Most interestingly, there is currently a lack of a direct and strong correlation between these neuroscientific findings and real-life behavior as well as cognition. However, in the context of modern plasticity research, we must take considerably more account of the fact that the brain can adapt and change anatomically and functionally through practice and learning ^5, 8^. Therefore, it could be possible that male and female brains might change their structure and functions because of their different experiences and because they are exposed to different social environments. Thus, the brain’s anatomical and functional sex/gender differences found so far can also be modulated by experience and not entirely by sex-related genetic influences. However, it is also possible that genetic, hormonal, and social influences interact in a currently unknown manner in forming brain and behavior. In light of these influences on the development of the human brain, a new area of sex/gender research could be established. We should consider the human brain more as a particularly adaptable organ that allows us to adjust to different environments and cultures.
